# Relationship between speaking English as a second language and agitation in people with dementia living in care homes: Results from the MARQUE (Managing Agitation and Raising Quality of life) English national care home survey

**DOI:** 10.1002/gps.4786

**Published:** 2017-10-03

**Authors:** C. Cooper, P. Rapaport, S. Robertson, L. Marston, J. Barber, M. Manela, G. Livingston

**Affiliations:** ^1^ UCL Department of Old Age Psychiatry, Division of Psychiatry London UK; ^2^ North Thames CLAHRC London UK; ^3^ UCL Department of Statistical Science Gower Street London UK; ^4^ UCL Department of Primary Care and Population Health and Priment Clinical Trials Unit UK

**Keywords:** agitation, care home, dementia

## Abstract

**Objective:**

As not speaking English as a first language may lead to increased difficulties in communication with staff and other residents, we (1) tested our primary hypotheses that care home residents with dementia speaking English as a second language experience more agitation and overall neuropsychiatric symptoms, and (2) explored qualitatively how staff consider that residents' language, ethnicity, and culture might impact on how they manage agitation.

**Methods:**

We interviewed staff, residents with dementia, and their family carers from 86 care homes (2014–2015) about resident's neuropsychiatric symptoms, agitation, life quality, and dementia severity. We qualitatively interviewed 25 staff.

**Results:**

Seventy‐one out of 1420 (5%) of care home residents with dementia interviewed spoke English as a second language. After controlling for dementia severity, age, and sex, and accounting for care home and staff proxy clustering, speaking English as a second language compared with as a first language was associated with significantly higher Cohen‐Mansfield Agitation Inventory (adjusted difference in means 8.3, 95% confidence interval 4.1 to 12.5) and Neuropsychiatric inventory scores (4.1, 0.65 to 7.5). Staff narratives described how linguistic and culturally isolating being in a care home where no residents or staff share your culture or language could be for people with dementia, and how this sometimes caused or worsened agitation.

**Conclusions:**

Considering a person with dementia's need to be understood when selecting a care home and developing technology resources to enable dementia‐friendly translation services could be important strategies for reducing distress of people with dementia from minority ethnic groups who live in care homes.

## 1 INTRODUCTION

Around 850 000 UK people live with dementia, and this is expected to be over 1 million by 2025.^1^ The number of people from Black and Minority Ethnic groups (BME) with dementia in England is projected to increase 7‐fold in the next 40 years.^2^ At least two thirds of care home residents have dementia.^3^ People with dementia from some BME groups are less likely to move to a care home than those from the white majority population.^4^ Possible explanatory factors include expectations among family carers that services will be culturally inappropriate (in terms of language and food), and a greater sense of filial piety and obligation.^5^ Care home staff have reported challenges providing culturally appropriate care, for example appropriate diets and translation services, and in day to day communication.^6^, ^7^ With the number of BME care home residents with dementia projected to increase due to demographic trends, it is important to consider how care homes can best provide culturally competent care.

In people with dementia, language impairment can impede functioning and effective communication, resulting in disruptive behaviour,^8^ due to unmet needs or frustration.^9^ Residents with dementia may become agitated if people use language beyond their comprehension or they are not understood when communicating.^10^ Language impairment may be especially challenging for people whose first language is not the local language, in whom cognitive decline can cause regression to the primary language, and loss of second language abilities.^11^

The MARQUE (Managing Agitation and Raising Quality of life) study includes the largest ever study of care home residents with dementia, the first to compare experiences of people with dementia who do and do not speak English as a first language. As not being a native speaker may lead to difficulties communicating with staff and other residents, we tested our primary hypothesis that care home residents with dementia speaking English as a second language experienced more neuropsychiatric symptoms, including agitation than those speaking English as a first language. Our secondary hypothesis was that compared with those speaking English as a first language, care home residents with dementia speaking English as a second language had more severe dementia, because they entered care homes later in the illness due to a range of barriers to access or family structure allowing them to be supported at home. We also interviewed care home staff qualitatively to explore how they consider residents' language, ethnicity, and culture to affect how they manage agitation symptoms.

**Key points**

- The MARQUE study is the first to compare experiences of people with dementia living in care homes who speak English as a second language to those who speak English as a first language.
- People with dementia living in care homes who speak English as a second language experienced more agitation and overall neuropsychiatric symptoms than those who spoke English as a first language, after controlling for dementia severity.
- Staff narratives illustrated how linguistic and culturally isolating being in a care home where no residents or staff share their culture or language could be for people with dementia, and how this sometimes increased agitation.
- Considering a person with dementia's need to be understood when selecting a care home and developing technology resources to enable dementia‐friendly translation services could be important strategies for reducing distress of people with dementia from minority ethnic groups who live in care homes.

## 2 METHODS

Harrow (14/LO/0034) and London (Queen's Square) (14/LO/0697) National Research Ethics Service (NRES) committees approved the quantitative and qualitative studies respectively. All staff and family carer participants gave informed consent before participating.

### 2.1 Setting and sampling

We recruited care homes across England, of each provider type (voluntary, state, and private), care provision (nursing, residential), and urban/suburban and rural locations, from July 2014 to October 2015.

### 2.2 Procedures

We recruited homes through third sector partners, NHS trusts, Care England newsletter, and the NIHR Clinical Research Network. We divided care homes into clusters, defining clusters as units within care homes. Most units comprised 1 whole care home, but where care homes operated as distinct units with discrete staff groups (for example, residential care and nursing care units operating as distinct entities), 1 care home was considered as 2 or 3 units. If staff cross‐covered between units, we defined this as 1 cluster.

We sought care home managers' agreement for their home's inclusion. In included homes, all consenting regular staff who provided hands‐on care were asked to complete measures. A senior staff member identified residents with a dementia diagnosis and for others completed the Noticeable Problems Checklist^12^ with care home staff to detect residents with undiagnosed probable dementia. We asked the paid carer working most closely with each resident with dementia, and their family carer if they visited at least once a month to complete proxy measures. For the qualitative interviews we purposively selected from the 86 care homes where quantitative data were collected. After initial contact with care home managers, we approached individual staff members who were involved in the day to day, “hands‐on” care of participating residents to complete proxy measures. We used purposive sampling to ensure that we interviewed staff of both sexes, differing age, ethnicity, nationality, and with different roles and experience.

### 2.3 Quantitative measures

Trained research assistants interviewed staff in private care home rooms. We interviewed family carers in their preferred location: the care home, their own home, or the researcher's office.

We asked managers about care home type (residential care, nursing care, or both), whether it was dementia specialist (staff had specific dementia training), and registered with regulatory authorities as providing care to people with dementia. We recorded residents' demographic information, including whether they spoke English as a second language and ethnic group; staff completed the following proxy measures:

1. The Cohen‐Mansfield Agitation Inventory (CMAI) is a 29‐item informant questionnaire with construct validity and reliability to measure agitation in people with dementia in care homes (32;33). Each item scores from 1 meaning “never” to 7 “several times per hour”. The score sums individual items. A score of >45 is usually regarded as clinically significant agitation (34).
2. The DEMQOL proxy is a responsive, valid, and reliable interviewer‐administered measure of quality of life in people with dementia (35;36).
3. Staff gave information so the researcher could rate dementia severity using the Clinical Dementia Rating (CDR) (37). This is reliable and valid (38). It is used to rate performance in Memory, Orientation, Judgment and Problem solving, Community Affairs, Home and Hobbies, and Personal Care. This information was used to classify dementia severity as very mild, mild, moderate, or severe.
4. The Neuropsychiatric Inventory (39) is a validated instrument with 12 domains of neuropsychiatric symptoms, including agitation. Each domain scores between 0 and 12 with higher scores meaning increasing severity. A summed scored is calculated for total neuropsychiatric symptoms (14).

We asked relatives visiting residents at least monthly to complete the DEMQOL‐proxy (36).

### 2.4 Qualitative interviews

PR conducted qualitative interviews in private rooms in care homes with staff who gave written, informed consent. We developed our semi‐structured interview schedule around our study aim to understand how staff managed agitation, using research literature, consultation with family carers of people with dementia, and research team expert opinion. We elicited staff perceptions using open‐ended questions and revised questions iteratively, exploring issues raised. We continued interviewing until we reached theoretical saturation. Interviews were audio‐recorded and transcribed verbatim.

### 2.5 Analysis

We used Stata version 14 for all quantitative analyses (40). Characteristics of care homes and people with dementia are summarised using frequency (%), mean (standard deviation), or median (interquartile range) as appropriate. To investigate our hypotheses, we used random effects models to account for care home/unit clustering and clustering by paid carer, as some paid carers provided information about multiple residents in the home. We adjusted for resident's age, sex, dementia severity, and care home type (residential/nursing/both, dementia specialist, dementia registered).

We used NVivo software for qualitative data analysis and took a thematic analytic approach.^13^ PR and a second, independent rater (CC) systematically coded the transcripts into meaningful fragments and labelled these initial codes. Discrepancies were discussed and resolved. PR and CC then organised the data into preliminary themes. We discussed the coding frames within the team using the constant comparison method to identify similarities and differences in the data.

## 3 RESULTS

### 3.1 Quantitative

Eighty‐six out of 114 (75.4%) of the care homes we contacted participated. Of the 28 who did not participate, 21 were nursing or mixed nursing and residential and 7 residential only. Twenty‐seven declined to participate, and 1 was excluded as they were taking part in another research project. We recruited 86 care homes; 7 homes were divided into >1 cluster, totalling 18 clusters. The sample, therefore, was 97 clusters.

Seventy‐one out of 1420 (5%) care home residents with dementia in our study spoke English as a second language; 37 (38%) of care home units were home to residents speaking English as a second language: 23 were home to only 1 resident, and the remainder were home to between 2 and 5 residents speaking English as a second language.

Tables 1 and 2 show the demographic, illness, and care home characteristics of residents speaking English as a first or second language, including information on missing data. As hypothesised, agitation and neuropsychiatric symptom levels were higher in those speaking English as a second language relative to those who were native speakers. After controlling for dementia severity, resident age, and sex and accounting for care home and staff proxy clustering, speaking English as a second language was associated with significantly higher CMAI (adjusted difference in means 8.3 (95% confidence interval [CI] 4.1 to 12.5) and neuropsychiatric inventory (4.1, 95% CI 0.65 to 7.5)) scores compared with those who were native speakers. These results were unchanged by controlling for care home type and dementia registration or specialism (Table 2) (8.4 [95% CI 4.2 to 12.6] and 4.1 [95% CI 0.64 to 7.5], respectively). For the CMAI (standard deviation 18.3), the effect size was 0.45.

**Table 1:** Resident demographic and illness characteristics of residents with dementia speaking English as a first or second language

		N (%) or median (IQR) in residents who did not speak English as a first language	N (%) or median (IQR) in residents who speak English as a first language
Age mean (SD) *Base population:*		82.7 (1.2) *n = 69*	85.0 (0.2) *n = 1327*
Female sex *Base population:*		38 (53.5) *n = 71*	939 (69.6) *n = 1349*
Ethnicity	White British/Irish	5 (7.0)	1293 (96.4)
	White other	30 (42.3)	15 (1.1)
	Asian	11 (15.5)	2 (0.2)
	Black	10 (14.1)	20 (1.5)
	Mixed/other	15 (21.1)	11 (0.8)
	*Base population*	*n = 71*	*n = 1341*
Dementia severity	Mild or very mild	19 (27.1)	400 (29.7)
	Moderate	22 (31.4)	444 (33.0)
	Severe	29 (41.4)	501 (37.5)
	*Base population*	*n = 70*	*n = 1345*
Neuropsychiatric inventory total score *Base population:*		14 (6,27) *n = 69*	9 (3,20) *n = 1322*
Cohen‐Mansfield Agitation Inventory *Base population:*		47 (38,61) *n = 69*	40 (33,54) *n = 1316*
Staff proxy DEMQOL score Base population:		*104 (95,109)* *n = 70*	*104 (95,111)* *n = 1343*
Family carer proxy DEMQOL score Base population:		*96 (83,107)* *n = 49*	*102 (91,109)* *n = 956*

**Table 2:** Demographic and illness characteristics of residents speaking English as a first or second language by care home type

		N of care home units in MARQUE study	N (%) residents speaking English as second language
Care home type	Residential	*12 (13%)*	*16 (3.0%)*
	Nursing	*39 (42%)*	*11 (5.1%)*
	Both	*41 (45%)*	*44 (6.5%)*
Dementia specialist	Yes	*41 (45%)*	*28 (4.5%)*
	No	*51 (55%)*	*43 (5.4%)*
Dementia registered	Yes	*81 (88%)*	*66 (5.1%)*
	No	*11 (12%)*	*5 (4.1%)*

Staff rated the quality of life of people speaking English as a first and second language similarly, while there was a trend towards lower family carer proxy ratings for people speaking English as a second language (Mann Whitney *U* test *z* = −1.9, *P* = 0.06). Contrary to our hypothesis, levels of dementia severity of residents speaking English as a first or second language were very similar (Table 1).

### 3.2 Qualitative

PR interviewed 25 staff in 6 care homes; London (4 homes), Kent (1 home), and Cambridge (1 home). Five of the care homes were privately run, and 1 was run by a charity. Three were nursing homes, 2 residential homes, and 1 provided residential and nursing care. Table 3 summarises staff socio‐demographic and employment status.

**Table 3:** Socio‐demographic and employment status of staff qualitatively interviewed

Socio‐demographic	Category	Care staff *n* (%)
Sex	Female	17 (68)
	Male	8 (32)
Ethnicity	Asian or Asian British	6 (24)
	Black or Black British	6 (24)
	White British	6 (24)
	White other	5 (20)
	Mixed other	2 (8)
English as first language	No	13 (52)
	Yes	11 (44)
	Not known	1 (4)
Staff role	Care assistant	9 (36)
	Manager/deputy manager	5 (20)
	Team leader	7 (28)
	Activities coordinator	2 (8)
	Nurse	2 (8)
Shift pattern	Days	18 (72)
	Days and nights	7 (28)
Length of service	Less than 1 year	4 (16)
	1 to 5 years	13 (52)
	6 to 10 years	8 (32)
Nursing qualification	Yes	10 (40)
	No	15 (60)

We identified 3 main themes. These were language barriers increase resident's agitation and staff and resident's distress, difficulties in meeting residents' unmet cultural needs, and overcoming barriers—finding shared language and understanding. We illustrate these themes with quotes later.

#### 3.2.1 Language barriers increase resident agitation and resident and staff distress

Several staff described how residents unable to communicate in English often became frustrated and agitated, when they could not express themselves or if they were unable to understand what was happening, for example during personal care:

Sometimes he's speaking his own language as well, which we don't understand, and he's crying, and it's difficult when we don't understand his language…, you know? (Female care assistant 1, CH1)

Staff described how residents had lost their English because of the dementia and how frustrating it was when struggling to respond and reduce distress:

Because of the dementia, they tend to become like a child again, they go back to their first or their original…. language. What they start saying is something that nobody understands, and that is dementia, they become a kid, a child, and .... I go, can you just say it in English so we can know how to help you. (Male senior carer, CH1)

#### 3.2.2 Difficulties in meeting residents unmet cultural needs

Staff highlighted how they sometimes struggled to connect with, understand, and respond to residents' cultural needs. A care assistant described how a resident's agitation seemed to relate to an unmet spiritual need:

You know, maybe in his young age it was used to pray at this time, I don't know, and that's it, they register that in their mind. And of course, because what do we do? Do we pray with him? Sometimes, you know, I do. (Male care assistant 2, CH1)

It was sometimes unclear how well information about residents' social and cultural backgrounds was shared and used within homes:

I saw her care plan, where she worked, she was born in India, she was brought up for 35 years in Bombay, she speaks Hindi, I've never spoken to her in English, we together speak Hindi, then my manager was asking, can she speak Hindi? She was surprised. (Female senior carer, CH3)

#### 3.2.3 Overcoming barriers—finding shared language and understanding

Staff recounted how agitated residents became calmer and more engaged when staff fortuitously shared their language or culture, or a friend or family member visited:

…her daughter was there, …then the menu was there, she said, I'm fed up with this food; then I asked her in the Indian language, her daughter doesn't understand because she was born here and brought up here, but her mother speaks it, then I gave her so many menu lists , …her favourite dishes are masa dosa and pani poori. (Female senior carer, CH3)

She tells us she knows English, but sometimes she forgets the English. She's always telling the Italian, so we don't... and she's deaf as well. Sometimes she doesn't understand what we are telling, but when [her Italian‐speaking friend] comes, when she speaks in Italian, she sometimes calms down. You know, she speaks to her. (Female senior carer 2, CH2)

Finding a staff member speaking the same language could be instrumental to resolving an episode of agitation:

I just want him to calm himself down, so... and if he doesn't listen to me, There's a lady who works here, she speaks the same language as him, so it is easy for him as well to understand her, so I just call her and say, oh, can you please help? (Female care assistant 1, CH1)

Other staff explained that they would try and find creative ways to communicate, by learning a few shared words or using non‐verbal approaches:

You try to talk… I mean, we ask the family the translation of the language, so we try to remember those words, language, and that's how we communicate with them. And we will rub his back and then try to calm him down. (Female care assistant, CH3)

…sometimes he points, like, you know, stomach. If you ask him, like, what's wrong with you, he will speak in his language, but he will point. (Female care assistant 1, CH1)

## 4 DISCUSSION

We confirmed our primary hypothesis that care home residents with dementia speaking English as a second language experienced more agitation and neuropsychiatric symptoms than those speaking English as a first language. These differences were not because they were living in the care homes later in their illness than those speaking English as a first language, because the severity of their dementia was not greater than those who spoke English as a first language. Most of the residents speaking English as a second language had clinically significant agitation levels, and the effect size we found (0.45) suggests that the increase in agitation in this group is likely to be clinically important.

In our qualitative interviews, staff spoke of the difficulties caring for residents with dementia when they did not share a language. Where possible, they found staff or relatives with the appropriate language skills, and otherwise managed as best they could through non‐verbal communication or learning a few words of the resident's language. Without a shared language, agitation was more difficult to manage and resolve. None of the staff mentioned access to professional translators, other cultural or language resources, or training they had received.

We have previously reported from the MARQUE study that agitation is associated with lower life quality.^14^ Family carers rated quality of life of residents speaking English as a second language lower, relative to those speaking English as a first language. For staff proxy raters, we did not find this difference. As family carers are more likely to share a language and culture with the resident compared with staff, this could indicate that staff underestimate the extent or impact of agitation where there are language or cultural barriers. We hypothesised that agitation and neuropsychiatric symptoms would be greater in people speaking English as a second language because there would be more language barriers preventing them from living well with dementia and receiving good care. Language barriers have been cited previously as a cause of reduced satisfaction with health or social care services.^15^

Interventions have successfully increased person‐centred care in care homes, through training carers to increase the quantity and quality of their verbal communication with residents, especially around personal care (ref Bourgeois). It was clear from staff narratives that residents with dementia speaking no English, who were losing their English skills or came from minority cultural groups, were often linguistically and culturally isolated, unable to routinely communicate through a common language, unless a staff member on shift fortuitously shared their language. There is evidence that BME groups have better mental health when living in areas with higher proportions of people of the same ethnicity.^16^ Care homes with language or cultural specialisms are rare, but where they exist, may be less isolating and comforting for residents and their relatives. More may develop as the older English BME population increases in size. Face‐to‐face interpreting services are expensive and tend in our clinical experience to be reserved for appointments with health or social care professionals. Where they are used more frequently, it is often in response to very severe agitation. Online interpreting services or other technology solutions could reduce language barriers in care homes and, together with asking families to provide staff with a few written words of basic vocabulary and training staff to understand and address unmet socio‐cultural needs, may be more feasible in the current climate of austerity. Considering whether staff speak the language and planning their shifts and using local cultural as well as individual resources could improve communication for some residents.

We cannot determine causality direction: families of participants speaking English as a second language might have had a particularly high threshold for deciding care at home was untenable, and thus this group may have had high levels of agitation at care home entry. This could explain our findings as opposed to or additional to agitation arising from greater communication difficulties experienced by non‐native speakers once in the care homes. We know that BME family carers are less likely to move a relative to a care home^4^ but did not find that those in the care home had more severe dementia.

MARQUE is the largest national care home survey, but our sample was not designed to be representative. We did not evaluate English language skills of residents who were non‐native speakers. These probably varied. We used language rather than ethnicity as primary outcome, as fitted our hypotheses, but these are closely related (Table 1). We cannot distinguish the impact of language, ethnicity, and culture in this study.

## 5 CONCLUSIONS

Care home residents with dementia who were non‐native speakers experienced more agitation and neuropsychiatric symptoms than native speakers. Staff narratives described how isolating being in a care home where no residents or staff share your culture or language could be for people with dementia, and how this sometimes increased agitation. Considering a person with dementia's need to be understood when selecting a care home and developing dementia‐friendly translation services could reduce distress for these residents. With numbers of BME people with dementia projected to rise, these are urgently needed.
